# Bapedi traditional healers in the Limpopo Province, South Africa: Their socio-cultural profile and traditional healing practice

**DOI:** 10.1186/1746-4269-10-4

**Published:** 2014-01-10

**Authors:** Sebua S Semenya, Martin J Potgieter

**Affiliations:** 1 Department of Biodiversity, University of Limpopo, Private Bag X1106, Sovenga 0727, South Africa

**Keywords:** Bapedi, Limpopo Province, Profile, Plant use, Traditional healers

## Abstract

**Background:**

Bapedi traditional healers play a vital role in the primary health care of rural inhabitants in the Limpopo Province, South Africa. However, literature profiling their social and demographic variables, as well as their traditional healing practices is lacking.

**Methods:**

Convenience sampling were used to identify and select two traditional healers from 17 municipalities (resulting in 34 healers being used in this pilot survey) of the Limpopo Province in South Africa. Information on the social and demographic variables, and traditional healing practices of these healers was gathered from January 2013 to July 2013, using a semi-structured questionnaire, supplemented by field surveys for plant identification and collection used in the preparation of remedies.

**Results:**

Males constituted nearly two-thirds of the participants. Forty eight percent of them became healers through the mentoring of another healer, while 38% acquired their traditional healing knowledge from parents and 14% from grandparents. In contrast to this, 62% of the females obtained theirs from their parents, 30% from fellow traditional healers, and 8% from grandparents. A total of 154 plant species were indicated as used by healers in the treatment of 52 health-related problems. A vast majority (89%) of these practitioners reported that prepared herbal remedies do expire, which is a temperature-dependent process. Determinations of the efficacy of remedies by most healers (67%) were via consultation with ancestors (90%). This study also found that none of the interviewees had any knowledge of provincial or national environmental legislation.

**Conclusions:**

The current study has shown that Bapedi traditional healers could play a leading role in both the preservation of indigenous knowledge and the primary health care sector. However, of concern is the traditional methods (via consulting ancestors) employed by most of these healers in determining efficacy of remedies, thus indicating a need for a scientific investigations to establish their safety and effectiveness. Equally, there is a need to educate traditional practitioners’ regarding the significance of various conservation legislations in their traditional healing. By addressing these, the national and provincial legislators, medical fraternity as well as environmental agencies will be able to better integrate them in primary health care systems and environmental management.

## Background

Due to high levels of poverty traditional medicines are considered essential for the physical and mental welfare of especially rural black households in South Africa, with more than 60% of all healing taking place outside the formal western-styled medical system [1]. Hoareau and DaSilva [2] stated that traditional medicine in several developing countries, incorporating local traditions and beliefs, is still the mainstay of primary health care; where modern health care facilities are either sparsely located or non-existent [3]. It is well-documented [4,5], that in the rural areas of South Africa, traditional healers operate in close proximity [6], and association [7], with the community members to treat various diseases and ailments.

Traditional healers are established health care providers within their respective communities [8]. Indeed Van Rensburg et al. [9], expand on this definition by stating that a traditional healer as someone who is recognized by the community in which he/she lives as competent to provide primary health care. These authors further stated that such a person utilise plants, animals and mineral substances together with methods based on the social, cultural and religious background, as well as prevailing knowledge, attitudes and beliefs for the physical, mental and social well-being of the community. Traditional healers are generally divided into two categories: Those that serve the role of diviner-diagnostician (or diviner-mediums), and those who are healers or herbalists [10].

Diviners are experts at applying diagnostic criteria [11]; they not only define the illness, but also its origin and reason in terms of African belief systems [12]. According to Gumede [13], diviners identify the origin and reason and prescribe an appropriate plant- and animal-based treatment for the affliction through spiritual means [10]. Chavunduka [14] reported that diviners communicate with spirits when in a state of possession, conveying the demands of ancestors and reasons for their dissatisfaction. According to Karim et al. [12], diviners’ speciality is divination within a supernatural context through culturally-accepted medium-ship with ancestral spirits. They are the most important intermediaries between humans and the supernatural. Unlike herbalist, no one can become a diviner by personal choice [12]. As this is an ancestral call, diviners regard themselves as servants of the ancestors [9].

Meissner [15] reported that herbalist practice the art of healing. Although this profession tends to run in families, Van Rensburg [9] indicated that the desire to become a herbalist is an individual’s choice and therefore, the profession is freely accessible to anyone [12]. The World Health Organisation [16] regards a herbalist as an ordinary person who have acquired extensive knowledge of medicinal plant use, and who do not, typically, possess occult powers. They are usually male, and are often selected and mentored by an established practitioner [13]. Herbalists are expected to diagnose and prescribe medicines for everyday ailments and illnesses; prevent and alleviate misfortune or evil; provide protection against witchcraft, and to bring prosperity and happiness [16].

Traditional healing has always been a component of health care in African countries, and the contributions of healers to primary health care sectors are well known in some parts of the African continent. For instance, in the Chiawa area of Zambia, Ndubani and Hojer [5], interviewed 23 healers about their knowledge, practices, and the use of indigenous plants in the diagnosis and treatment of sexually transmitted infections. These authors concluded that healers should be integrated in to the sexually transmitted infections control scheme in Zambia. However, they further noted that the government should provide them with the necessary health-related information and financial and material support.

In South Africa, the roles and profiles of traditional healers of some cultural groups has been documented. For example, Puckree et al. [17] studied the socio-cultural profile of Zulu traditional healers (diviners and herbalists) in Durban, KwaZulu-Natal Province. They investigated the following issues; the role if healers, number of patients that consulted traditional healers, the types of conditions treated, as well as the frequency of consultations. Their findings indicated that, a considerable number of patients consulted a traditional healer as a first choice for both physical and mental ailments, and diviners being the most popular type of healer (visited for these ailments). Bereda [18] reported on the role of VhaVenda traditional healing as a health care delivery system in the Vhembe district, Limpopo Province. The results of this study indicated that a larger proportion of male diviners are mostly consulted for the treatment of a variety of ailments including asthma, diabetes mellitus, hypertension, tuberculosis and sexually transmitted infections. Bereda [18] mentioned that since a significantly large number of patients consult traditional healers for a variety of ailments, including potentially life-threatening conditions, health care professionals should be proactive in integrating traditional healing with westernized practices in order to promote health care for all.

Another study in Vhembe district, but conducted by Mabogo [19], emphasized the role of VhaVenda traditional healers and their *materia medica*. This study found that both herbalist and diviners are instrumental in the treatment of various human diseases, including sexually transmitted infections and tuberculosis. However, diagnosis of ailments was primarily based on the presentation of symptoms, and sometimes traditional rituals. Regarding their herbal remedies, Mabogo [19], noted they prepare them from more than 50 plant species. According to this author, documentation and understanding of traditional healing practice’ knowledge is very crucial, as it might provide solutions to future challenges of diseases, and contribute to the conservation of useful wild plants.

The current study will therefore, further contribute towards documenting and describing the diverse spectrum of individuals (only herbalists), but among the Bapedi, involved with the practice of traditional medicine in the Limpopo Province of South Africa. It is envisaged that this information will provide a clearer picture of the socio-cultural profile and traditional healing practice of Bapedi health care providers who play a crucial role in the primary health care of especially rural areas.

## Methods

### Study area and study population

**Figure 1:**
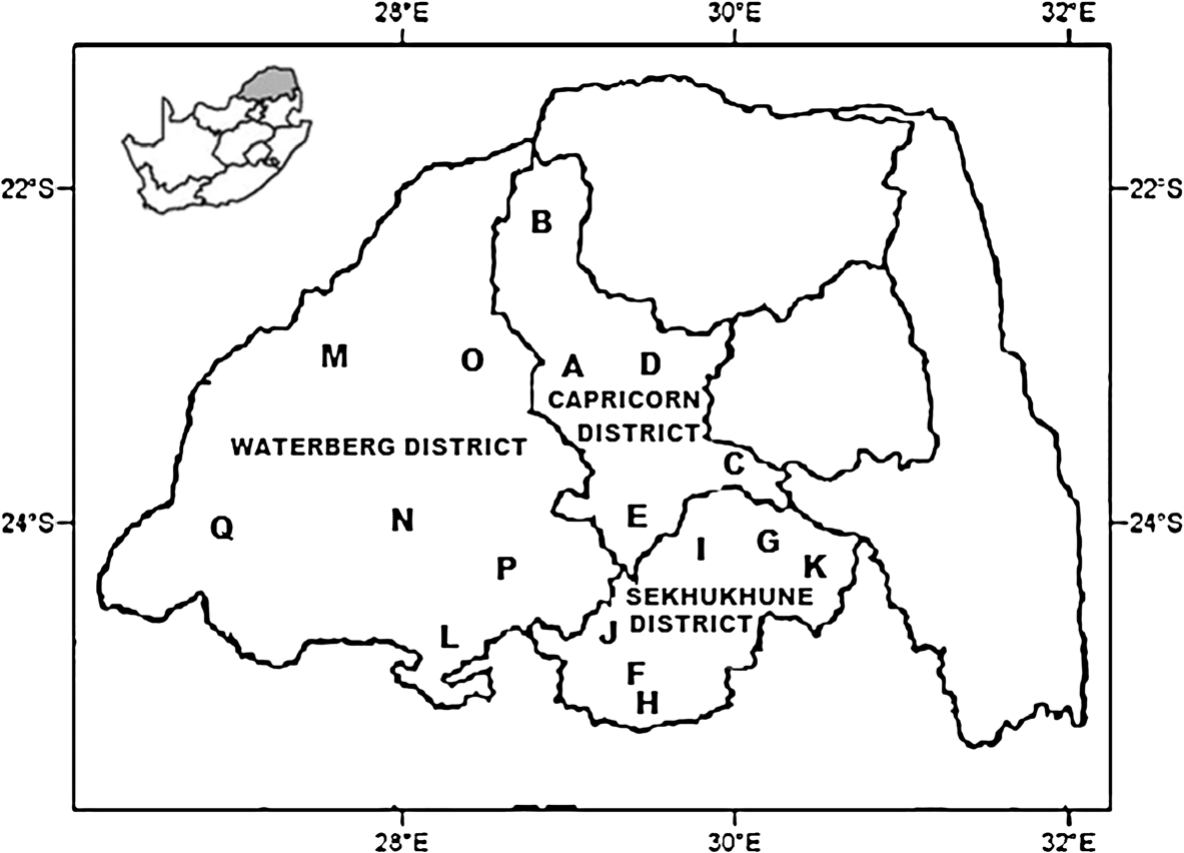
**Study area: Capricorn, Sekhukhune and Waterberg Districts, Limpopo Province, South Africa.** A to Q (in Figure 1 and Table 1) designates the involved municipalities.

The study was conducted in the three districts (Capricorn, Sekhukhune and Waterberg) (Figure 1) covering 17 local municipalities (Table 1) of the Limpopo Province, South Africa. The surveyed districts are the cultural home of the Bapedi, who resides primarily in the central, southern and western parts of the province, where they constitute the dominant ethnic group [20]. The Bapedi people are of Basotho descent, who migrated south from the Great Lakes more than 500 years ago [21]. The vegetation in these districts was classified by Acocks [22] as a semi-arid savanna, characterized by a mixture of trees, shrubs, herbs and grasses [23]. This type of vegetation has provided a diverse flora with rich medicinal plants that the traditional healers of the study areas have used to treat many illnesses.

**Table 1:** Districts and local municipalities included in this study

**Capricorn district**		**Sekhukhune district**		**Waterberg district**	
Aganang	**A**	Elias Motsoaledi (Greater Groblersdal)	**F**	Bela-Bela	**L**
Blouberg	**B**	Fetakgomo	**G**	Lephalale	**M**
Lepelle-Nkumpi	**C**	Groblersdal	**H**	Modimolle	**N**
Molemole	**D**	Makhuduthamaga	**I**	Mogalakwena	**O**
Polokwane	**E**	Ephram Mogale (Greater Marble Hall)	**J**	Mookgophong	**P**
		Tubatse	**K**	Thabazimbi	**Q**

### Socio-cultural and traditional healing practice surveys, and data collection

This study was conducted from January 2013 to July 2013. Prior informed consent was obtained from all participants, in line with the requirements of the University of Limpopo’s ethical prescriptions. Thirty four traditional healers (2 per local municipality) were identified and selected from the municipalities mentioned below (Table 1) by convenience sampling; i.e., with the assistance of fellow healers and villagers. This sampling technique was utilized due to the numerous advantages it provides. For instance, it is extremely fast, easy, readily available, and cost effective. However, the results from a study conducted with such a sampling technique cannot be generalised to the population as a whole or cannot be an accurate representation of the population [24].

A semi-structured interview form was used to obtain information from healers regarding social and demographic variables, as well as some information related to their traditional healing practices. The questionnaire addressed the following issues; (i) demographic profile: age, gender and educational level, (ii) questions dealing with traditional practices: history of becoming a healer, work style, diagnosis and treatment, (iii) aliments treated and remedy used, (iv) expiration of medicine, and (v) legislative and conservation-related matters. Traditional healers were interviewed (using Sepedi, a local dialect) independently from each other, in the confines of their consultation rooms.

### Plant collection and identification

Researchers collected medicinal plant materials from both home gardens and natural communal areas during organized tours while being accompanied by a traditional healer. The collected specimens were initially identified by their local vernacular names, while their taxonomic identification was done using the Larry Leach Herbarium of the University of Limpopo (UNIN). Collection numbers of species are presented in Additional file 1: Table S1.

### Data analysis and reporting

Descriptive statistics, such as percentages and frequencies, were used to analyse the data obtained from the questionnaire. The data was organised and analysed using the statistical program SPSS version 14.0, and in some cases Microsoft Excel.

## Results

### Gender, age and years in practice

The majority (62%, n = 21) of participating healers were males, with females constituting the rest (38%, n=13). Only 19% of the male participants were younger than 41, while 52% were between 41 and 50, 24% between 51 and 60, and just 5% older than 60. The largest proportion (62%) of females was between 51 and 60, with none older than 60 or younger than 30. Twenty three percent was between 41 and 50, and 15% fell within the 30 to 40 age category.

Fifty two percent of male healers have been in practice between 5–10 years, 43% between 11–30 years, and only 5% have more than 40 years’ experience. Forty six percent of female participants have been in practice for 6 to 10 years, and 54% for between 11 and 30 years.

### Level of education

**Figure 2:**
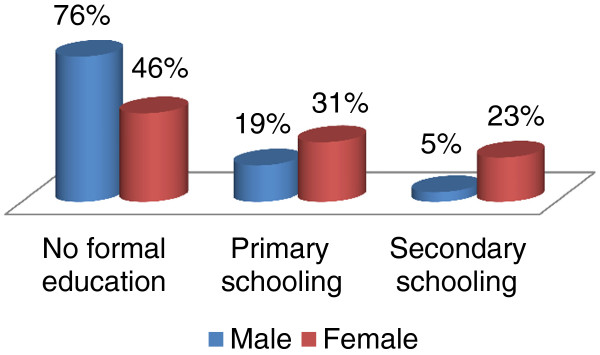
Level of education: a gender-based comparison.

The majority of males (76%), but less than half of the females (46%), in this study had no formal education. A larger proportion of females had primary school education (31% vs 19%) and secondary school education (23% vs 5%) than their male counterparts (Figure 2).

### Sources of traditional healing knowledge

Various sources of traditional healing knowledge exist among the Bapedi; such as fellow healers and family members. Most males (48% of total) acquired their healing knowledge from fellow traditional healers, 38% from their parents and 14% from grandparents. In contrast to this, 62% of the females obtained theirs from their parents, 30% from fellow traditional healers, and 8% from grandparents.

### Ailments treated, and used remedies (use plant part, methods of preparation and administration)

Fifty two health-related problems or ailments were found to be treated by Bapedi traditional healers in the poor rural areas of the Limpopo Province. Such problems/ailments included among others; abortion, appetite, asthma, blood clotting, blood purifier, body pain, breast cancer, chlamydia, circumcision wound, contaminated blood, depression, diabetes mellitus, diarrhoea, epilepsy, erectile dysfunction, eye infection, female infertility, goitre, gonorrhoea, HIV/AIDS, heart problem, hypertension, kidney problems, leukemia, low sperm count, malaria, measles, menstrual disorders, mental illnesses, nose bleeding, period pains, stomach complains, stroke, swelled legs, tonsils, tuberculosis and womb problems.

**Figure 3:**
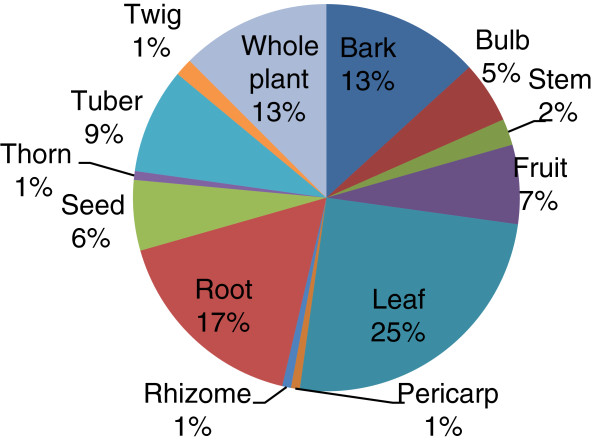
Plant parts used to prepare remedies.

Following symptomatic diagnosis, the above mentioned ailments were treated using plant-based remedies prepared from 154 species (Additional file 1: Table S1). Different plant parts including bark, bulb, fruit, leaf, pericarp, rhizome, root, seed, stem, thorn, tuber, twig and whole plant were utilised in the preparation of remedies. There was a distinct preference for leaf and root material as well as for the bark and whole plant (Figure 3). For increased efficacy traditional healers can combine different parts of the same plant during preparation of remedies. Similarly, substances such as cow milk, salt, Vaseline®, and soft porridge are mixed with remedies to enhance its effectiveness.

Remedies were prepared mostly via boiling (69%). However, other methods such as pounding (16%), burning (5%), macerating (4%), steaming and raw prescription (2% each), crushing, frying and squeezing (1% each), were also employed. Prepared medicines were administered in a variety of ways. For instance, medicines were administered/prescribed orally (as a liquid, via soft porridge, as a raw prescription or as smoke), nasally (plant parts were steamed or burned, and the resulting smoke/steam inhaled; topically: prepared remedies were applied by either a patient or healer on the affected body part/s); anally (a healer administered remedies via a bulb syringe). The disappearance or the improvement of symptoms reported by patients was perceived as independent verification of the effectiveness of a treatment.

### Expiration of medicine

Eighty percent of traditional healers reported that prepared remedies do expire, which is a temperature-dependent process; i.e., medicines expire quicker in hotter climates. Seventy five percent of respondents indicated that liquid medicine stored in a hot environment will expire within one week or even less, whereas those kept in cooler places can last for up to two weeks from their date of issue. The remaining 25% claimed that liquid medicine will expire within two to three days. Characteristics of expired liquid medicine can include any combination of the following; a tendency to change colour, coagulate into a paste, develop an odour or become extremely sour.

Pounded medicine on the other hand has a far longer shelf life and can remain effective for up to one year. The determining factor seems to be exposure to moisture. When expired, powdered remedies tend to either stick together or they won’t mix with water, even when shaken.

### Side effects and assessment of efficacy of medicine

Sixty percent of Bapedi healers claimed that their preparations were free of side effects. This was based on the fact that none of their patients reported side effects after treatment. Forty percent of healers indicated some preparations, especially for HIV/AIDS-related symptoms (dysentery and loss of appetite), and gonorrhoea (sexual dysfunction) has side effects.

The assessment of the effectiveness of the medicines is mostly based on the consultation (through performance of rituals) with ancestors, (deceased family members), with 90% of Bapedi healers claiming that their ancestors confirmed the effectiveness of medication. Ten percent of respondents noted that it is their treated patients (reported positive feedback) who validated the efficacy. However, 23% of the interviewees reported that patients with ailments related to HIV/AIDS returned for further treatment. In these situations healers either replenish the medication or refer the patient to a clinic or hospital. They hardly, if ever, send a patient to a fellow traditional healer for further treatment.

### Plant collection rituals

All healers perform rituals subsequent to harvesting plants; 90% of them doing so, as a means of expressing gratitude towards ancestors, whilst the remaining 10%, because of cultural norms.

When questioned on the efficacy of their medicine, 90% of the healers indicated that their ancestors confirmed the effectiveness of their remedies.

Bapedi healers believe in order to ensure efficacy of their medicine, plant parts must be collected by a person who has not had sexual intercourse for at least two days prior to collection. Most healers (79%) prefer to collect their own medicinal plants, as a safeguard against sexual impurity. In contrast to this preference a minority (21%) dispatch trainees for collections; however, they are carefully instructed on the custom of harvesting medicinal material.

### Harvesting plants

This study noted that it is customary not to re-fill the soil after harvesting underground parts or entire plants. The reasons forwarded were that re-filling the soil influence the effectiveness of harvested plants parts, worsen a patient’s illness and affect the healing progress of a patient. Bark was only collected from the side of the tree facing east; due to a belief that bark harvested from this side has the highest nutritional level of all sides.

### Legislative impacts

In terms of compliance to legislative requirements, this study found that none of the traditional healers had a permit to collect wild medicinal plants. They viewed the permit system as an obstacle to their practice. Furthermore, none of them has ever heard of the Limpopo Environmental Management Act (LEMA), which governs, amongst others, all aspects related to the collection, transport and relocation of plant species in the Limpopo Province. When made aware of this legislation, healers responded that environmental statutes have no bearing on their profession; as they view wild plants as common property.

## Discussion

### Gender, age and years in practice

In general traditional healing is a gender-based practice; although in some communities both men and women are equally involved in this profession [25]. In this study males constituted nearly two-thirds of the participants, which is in line with a number of other studies in the Limpopo Province. For example, Bereda [18] also noted the dominance of males in traditional healing in the Vhembe district; areas dominated by the VhaVenda tribe. Moeng and Potgieter [26] reported that male healers dominated the trade in medicinal plants in the Capricorn, Sekhukhune and Waterberg districts. This phenomenon might, even if only partially, be attributed to the fact that these males are generally involved in providing income for their families in rural areas. Moeng and Potgieter [26] further noted that the dominance of males could be due to that fact that the collection of medicinal plants may be physically risky for women, as most plants are found in mountainous areas. In the Western Cape Province of South Africa, Loundou [27] claimed that male dominance could be ascribed to the fact that some plants are located on private land making the collection of medicinal plants is too risky for women. In the Zegie Peninsula, North-western Ethiopia traditional knowledge in the family or community is mainly passed from the father to his first born son. It is therefore not surprising to find that males dominate the traditional healing profession [28]. Evidence such as this would support the notion that the Bapedi traditional healing industry might, in the near future, continue to be dominated by males.

Most healers are between 41 and 60 years. These results are very similar to that reported by Ndawonde [29] for Zulu traditional healers of KwaZulu-Natal, South Africa. He found that the majority of participant’s ages ranged from 45 to 54. Mintsa Mi Nzue [30] reported that the majority of male and female Xhosa healers, residing in the Western Cape Province of South Africa, were between 41 and 50. According to Ndawonde [29], the dominance of this age category is most probably due to the fact that they are considered to be responsible, and that most of them are in need of money to educate their children and support their basic livelihood needs. In light of the low life expectancy for South African males (45.1) and females (50.7) [31], the future prospects for the protection of indigenous knowledge might be at risk. This necessitates the urgent and rapid recording of indigenous knowledge related to this industry.

The small number of Bapedi traditional healers between 30 and 40 years of age, probably indicate a lack of interest for this profession, or perhaps symptomatic of a transitional society that is losing its cultural identity, as it westernise.

In accordance with Gessler et al. [32], this study found that both male and female healers started the traditional healing profession in their 20s or 30s. However, gender disparities are evident. Females, due to social responsibilities, enter the profession at an older age when compared to their male counterparts. Furthermore it was found that females do not occupy the profession as long as males, as they seem to retire at the age of 60; with some males continuing to practice well beyond the age of 60. Fifty seems to be the gender-divide as most of the males (71%) were between 30 and 50 years old, compared to 62% of the females being between 50 and 60.

According to Richter [33], the sum of all knowledge and practices, whether they are explicable or not, employed in the diagnosis, prevention and elimination of physical, mental, or societal imbalance, and relying exclusively on practical experience and observation are handed down, either verbally or in writing, from generation to generation. With regard to males, in this study, an even spread is apparent between young (<10 years’ experience) and the older and more established healers (>10 years). It can be argued that this balance between experienced and less experience individuals can contribute to the long term stability of this profession among the Bapedi.

### Level of education

This study found that a significant portion of the healers did not receive formal education (grade 1 to 12), with only a small portion attending school. This is in agreement with Mabogo regarding the Venda region [19], and Yineger and Yewhalaw in South-western Ethiopia [34]. However, the result of this study is not in agreement with other ethnobotanical studies conducted throughout South Africa. For instance, in KwaZulu-Natal, Puranswai [35] found that all Zulu traditional healers had attended school, while 20% even had a University degree or diploma. Likewise, Mintsa Mi Nzue [30] noted that half of the Xhosa traditional healers had attended secondary school, 35% primary school, and 3% a tertiary institution. Low level of education amongst Bapedi traditional healers compared to the above mentioned South African cultures is an indicative of fewer younger and older Bapedi healers, thus indicating a need to document their knowledge of *materia medica* before it get lost.

Richter [33] noted that nowadays educational skills are vital to traditional healers to empower them with particular competencies such as reading that might be important for counselling, and to understand the necessity to conserve and manage natural resources. Thus initiating programmes such as ABET (Adult Basic Education and Training) is important to empower not only Bapedi traditional healers, but all other traditional healers, with basic educational skills such as writing and reading. Basic education is therefore vital for healers to broaden their knowledge regarding conservation issues related to protected and threatened species, and medically-related issues such as diagnoses and treatment of ailments. Greater levels of education and consequently awareness could contribute in the long term sustainability of this highly important profession.

### Source of traditional healing knowledge

Forty eight percent of male traditional medical practitioners became healers through the mentoring of another healer. This trend was also reported by Cheikhyoussef et al. [36] for traditional healers of the Oshikoto region of Namibia. They found that most healers in this region obtained their healing knowledge from their fellow traditional healers. Normally indigenous knowledge of medicinal plant utilization is transferred from parents to children within a family [37]. The circumstances persuading established Bapedi traditional healers and the apprentice to interact are currently unknown. However, the possibility exists that some traditional healers might have been paid by their mentees for training.

Thirty eight percent of male traditional healers have acquired their traditional healing knowledge from parents and 14% from grandparents. Similarly, in the Saperas community of Khetawas, Jhajjar district, Haryana of India, male traditional healers also obtained their knowledge from parents and grandparents [38]. Among the Pedi, a culture exists where males are expected to be independent at an earlier stage. Therefore both parents might have trained them to become independent traditional healers and take advantage of the heavy reliance of rural communities on traditional healing services to make a living from such services.

The source of medicinal plants knowledge is the main contributor to the difference in knowledge and use between male and female traditional healers [25]. Most (62%) female Bapedi healers were trained by both parents to become traditional healers. Similar observations were reported in Ethiopia [39]. This is not surprising, as in African cultures daughters are usually more closely associated with both parents than sons. Through these close interactions, Bapedi females might have become interested and motivated by their parents to also practice this profession. Only a small percentage (30%) of females in this study obtained their traditional healing knowledge from fellow healers, thus reflecting the strong bond between daughters and parents in Bapedi culture.

### Ailments treated and used remedies (used plant parts, methods of preparation and administration)

A considerable number of health-related problems (52), treated by Bapedi traditional healers in the poor rural areas of the Limpopo Province strengthen the fact that traditional medicine and traditional health practitioners represent the first line of healthcare for the majority of people in this province [40]. Furthermore, the diversity of ailments treated is an indication that medicinal plants have a potential of satisfying the varied healthcare needs of poor villagers of the Limpopo Province. Most of the ailments treated by healers of the current study listed in Additional file 1: Table S1, including chlamydia [41], diabetes mellitus [42], diarrhoea [43], epilepsy [34], erectile dysfunction [44], eye infection [45], gonorrhoea [46], HIV/AIDS [35,47,48], hypertension [49], malaria [50], menstrual disorder [51], mental illness [52], and tuberculosis [53], are common amongst the healers of other ethnic groups in South Africa.

Interestingly, a South African study [47] indicated that some of the ailments treated by Bapedi healers, including sexually transmitted infections (chlamydia, gonorrhoea and HIV/AIDS), chronic diseases of lifestyle (diabetes mellitus and hypertension) as well as psychological ailments [18], are also commonly treated by traditional healers from other cultures. Thus, their documentation in the present study just goes further to buttress this. Bereda [18], found that in the Limpopo Province, professional nurses and doctors either utilise the service of traditional healers or refer their patients to them for the treatment of ailments such as mental illness, sexually transmitted infections, infertilities and erectile dysfunction. Continuing preferences of traditional healer’s health services over contemporary doctors are driven by a variety of factors including the faith people have in the healers’ herbal remedies [54]. Consequently collaboration between traditional healers and Western doctors regarding the treatment of some of human ailments will be a key to health care for all people of Limpopo Province and the rest of South Africa.

The current study further indicated that 154 plant species are being used by questioned healers to treat 52 health-related problems, which to some extent reflect the strength of Bapedi traditional medicine as it presents alternatives for the treatment of aliments. To the best of our knowledge the following two species; *Aloe angolensis* (appetite) and *Turraea obtusifolia* (blood purifier) are recorded for the first time as a treatment of the mentioned problems. New utilizations of these species by healers of the current study provide valuable contributions to the ethnobotanical records of South Africa and elsewhere.

Leaves were the morphological plant part most preferred in the preparation of remedies, followed by root and bark. This finding is in partial agreement to that noted by Cheikhyoussef and colleagues [36], in Oshikoto region, Namibia. They found that traditional healers residing in this region use a wide range of various plant parts (twig, bark, stem, tuber, pod and seed, among others), but root, leaf and even the whole plant play a significantly important role in the preparation of medicines.

Bapedi traditional healers prepared remedies from various plant parts mainly as water extracts based on single plant species. However, some preparations used as a treatment of blood clotting, blood purifier, chlamydia, diarrhoea, erectile dysfunction, female infertility, gonorrhoea, heart attack, HIV/AIDS, hypertension, malaria, tuberculosis and wound/general injury included species combinations for increased efficacy. Most of the species combinations were used to treat HIV/AIDS (Additional file 1: Table S1). This custom of employing multiple species is in line with western applications employing multiple therapeutic agents (cocktails) to combat the symptoms and progression of HIV/AIDS. This underscores the fact that Bapedi traditional healers understand the complex presentation of this debilitating disease.

Various extract preparation methods, such as boiling, pounding, burning, macerating, steaming, raw prescription, crushing, frying and squeezing, are respectively employed by Bapedi traditional healers. Most of these methods are very commonly used in the treatment of ailments in South Africa [19,48,55,56] and other African countries such as Ethiopia [57], and Kenya [58]. Similarly, routes (anal, oral and topical) of medicinal administrations used by healers of the current study are common elsewhere [34,59,60]. Occasionally, these healers administered medication rectally using a bulb syringe; in such cases they preferred to perform the administration themselves. The limited use of this method was not surprising as it was indicated that the procedure is very dangerous and is mostly performed by the more experienced healers. All interviewed healers agreed that incorrect dosages, especially in cases of an overdose, can be fatal. Generally, the disappearance or the improvement of symptoms reported by patients was perceived as independent indicators of a successful treatment of ailments.

### Expiration of medicine

Eighty percent of Bapedi traditional healers acknowledged that processed plant material do expire. The participants in this study identified temperature as the major contributor to the expiration of especially liquid medicine. They indicated that medicines stored in a hot environment have the potential to expire within a week or less, whereas those stored in cooler places can last significantly longer. Of interest is the fact that Bapedi healers and Nepalese [61], medicinal traders have similar discerning characteristics of expired liquid medicine. Among these the most prominent were any combination of the following; a tendency to change colour, become thick, has a rancid smell or become extremely sour. The reliance on empirical observations by Bapedi healers, as is probably the case in many other cultures, to determine the expiry date of any given prescription is far from optimal and thus warrants further investigation. This is in line with Griggs et al. [62] who noted that only a small number of species has been assessed for retention of activity over time, and that there is not enough scientific evidence available to serve as a decision-making framework regarding the expiration dates of ethno-medicine.

Pounded medicines were perceived to have a far longer shelf life and Bapedi traditional healers claimed that it could remain effective for up to one year. This claim is in agreement with findings of Blackburn [63], who reported that tablets and pounded medicine lasts longer than liquid medication. The determining delimiting factor for the shelve-life time of pounded medicine, according to the Bapedi healers is exposure to moisture, and characteristics of expired pounded medicine include sticking together or an inability to mix with/dissolve in water, even when shaken.

### Side effects and assessment of efficacy of medicine

Sixty percent of the traditional healers in this study claimed that their preparations are side effect free. Oyedemi et al. [64], also reported similar claims among the Xhosa traditional healers in the Eastern Cape Province of South Africa. These claims, by both Bapedi and the Xhosa healers are based on patient feedback. This approach to determine the presence or lack of side effects will remain a challenge as alternative medicine is mostly based on historical or cultural values, rather than on empirical evidence. This situation can be further confounded, in support of a report by Yineger et al. [65], it is considered that there might be an inability to; or a very low level of recognition with regard to adverse effects by healers; and absence of antidotes for those remedies might sometimes worsen the health problem of patients.

Van Wyk et al. [66], noted that side effects of remedies are known by experienced traditional healers. Our study confirms this observation for the Bapedi, with 40% indicating that some of their preparations have side effects. These included side effects for example of gonorrhoea (impotency/sexual dysfunction), as well as HIV/AIDS (dysentery and loss of appetite) preparations. According to Bapedi healers, these side effects are signs that the medicine is effective.

Sixty seven percent of Bapedi healers indicated that their herbal remedies are effective. This is determined by consulting ancestors (90%) or by patients’ positive responses (10%). Kamatenesi-Mugisha et al. [60] and Bhattarai et al. [67] noted that traditional healers use patients as a form of assessment of the efficacy of their medicine. The use of ancestors by Bapedi healers to confirm the efficacy of prescribed medicine are not surprising, as most African traditional healers believe that the ancestors have positive influences on medicinal plant prescriptions [14]. The main challenge facing the continued use of traditional medicine is proof that the active components contained in medicinal plants are safe and effective [68]. Despite the traditional methods employed by Bapedi healers in determining efficacy of remedies, scientific investigations are required to assure the medical field and an increasing knowledgeable public regarding the efficacy and safety of the use of medicinal plants as therapeutic alternatives.

### Plant collection and rituals

Seventy nine percent of Bapedi healers collected their own medicinal plants. This seems to be a general pattern as Kambizi and Afolayan [69] reported almost a similar finding in the Guruve district of Zimbabwe. They noted that it is only under certain circumstances, such as the unavailability of the healer, that an ordinary person could harvest plants on behalf of the healer. One reason for this preference to collect their own plant material is put forward by Nanyingi et al. [58] who noted that in the Samburu district, Kenya, healers’ preference to collect their own plants was to preserve the secrecy of plant habitat locations.

Ninety five percent of the participating Bapedi traditional healers practiced rituals prior harvesting plants, as a means of expressing gratitude to the ancestors. According to them this ensures that ancestors reveal the location of plants in the wild, and also guarantee that the medicine prepared from such species work effectively. Chavunduka [14] confirms this notion by stating that the ancestors play a role in transferring indigenous knowledge to healers so that they are able collect the right species, process it correctly and safely treat patients.

Sixty seven percent of Bapedi healers indicated that their herbal remedies are effective. This is determined by consulting ancestors (90%) or by patient’s positive responses (10%). This is in line with comments from Kamatenesi-Mugisha et al. [60] and Bhattarai et al. [67], who noted that traditional healers of Uganda and Nepal, respectively, use the treated patients as an assessment of the efficacy of their medicine. The use of ancestors by Bapedi healers to confirm the efficacy of prescribed medicine came as no surprise, as most African traditional healers believe that the ancestors have positive influences on medicinal plant prescriptions [14].

### Harvesting plants

When harvesting underground parts, Bapedi traditional healers did not re-fill the soil, because they believe it will worsen a patient’s illness. This is in accordance to the observation by Magoro [8] for Bapedi traditional healers in the Sekhukhune district. However, Kambizi and Afolayan [69] indicated that it is forbidden, among Shona traditional healers of Zimbabwe, not to refill the pit from which the roots are dug and that disregarding this would only worsen the illness. The custom of Bapedi healers of not re-fill the harvesting underground parts might result in plant water up-take reduction, plant carbohydrate reserve depletion and nutrient flow disruption or increase susceptibility to fungal attack which will eventually kill the species [70].

In this study the phenomenon of bark harvesting only from the side facing East, was also reported by Mabogo [19] for Vha-Venda traditional healers; a cultural group residing within the Limpopo Province. In a similar vein Ndawonde [29] reported bark harvesting by Zulu healers on both the east- and west-facing sides. Bapedi, Vha-Venda (eastern side) and Zulu (east and west-facing sides) traditional healers mentioned that bark harvested on these sides provide more healing power than the other sides. These healers believe that the east-blowing wind carry with it healing properties, and then comes back west, bringing the healing powers back.

### Legislative impacts

Current legislation requires the possession of a permit when plants are harvested from communal land. However, none of our participants had a permit, to collect medicinal plants from communal land, as they perceived it as an obstacle to their practice. This concept of limiting access to medicinal plants, is in contrast to the findings of Moeng and Potgieter [26] who noted that 63% of the *muthi* (medicinal and spiritual plant and animal material) traders (traditional healers included) didn’t see the need for such a permit; i.e., the long-term benefit of such a system. It is therefore recommended that any future implementation of the permit system should consider these driving-forces.

According to the Department of Environmental Affairs and Tourism [71] and the Convention on Biological Diversity [72], the lack of knowledge pertaining environmental legislation and conservation are key obstacles threatening the conservation of South African biodiversity. This study revealed that none of the interviewed traditional healers had any knowledge of LEMA [73]. This is understandable when viewed against the backdrop of educational levels or the lack thereof. In this study 95% of males and 77% of females, either had no schooling or only primary schooling. This in itself could limit the individuals’ ability to comprehend legislative documents, thereby impacting negatively on any conservation strategy. It is clear that education can play an important role in empowering them to manage their resources more effectively.

### Limitations of the study

The clearest limitation of this study was the low number of questioned traditional healers. It is worth mentioning that after an exhaustive literature search, as well as consultations with the relevant local government officials, we learned that currently there is no official documentation that indicates the total number of healers in either the studied municipalities or districts. Indeed, we concur with Espinosa et al. [74], that a low number of participants in an ethnobotanical study are not a true reflection that findings are representative of the population being investigated.

## Conclusions

This study has shown that Bapedi traditional healers could play a leading role in both the preservation of indigenous knowledge and the primary healthcare sector. Higher diversity of species used to treat an array of ailments is a reflection of the significant of plants in Bapedi traditional healing, and probably the role of Bapedi healers in the traditional primary health care sectors.

However, of concern is the traditional methods (via consulting ancestors) employed by most of Bapedi healers in determining efficacy of remedies, thus indicating a need for a scientific investigations to establish their safety and efficacy. Equally, there is a need to educate these healers regarding the significant of various conservation legislations in their traditional healing. By addressing these, we will be able to better integrate them in primary health care systems and environmental management.

Although the current study has achieved its aim of describing the diverse spectrum of some Bapedi healers and their traditional healing practice (such as plants used to prepare herbal remedies, and ailments treated) in the Limpopo Province; there is a great need to sample a larger proportion of healers in the studied districts and municipalities to verify the accuracy of the results.

## Competing interests

The authors declare that they have no competing interests.

## Authors’ contributions

SSS conceptualized the idea, collected the data and analyzed, and wrote the manuscript. MJP helped to finalise the manuscript. Both authors read and approved the final manuscript.

## Supplementary Material

Additional file 1: Table S1 Plant species, mode of remedy preparation and administration, as well as ailments treated by Bapedi traditional healers in the Limpopo Province. Click here for file
