# Supplementary material for: Bapedi traditional healers in the Limpopo Province, South Africa: Their socio-cultural profile and traditional healing practice
Source: J Ethnobiol Ethnomed. 2014 Jan 10;10:4. doi: 10.1186/1746-4269-10-4 (PMC3904471; doi:10.1186/1746-4269-10-4)
Supplement: Additional file 1: Table S1 — Plant species, mode of remedy preparation and administration, as well as ailments treated by Bapedi traditional healers in the Limpopo Province. [file 1746-4269-10-4-S1.docx]

| **Additional file 1: Table S1.** Plant species, mode of remedy preparation and administration, as well as ailments treated by Bapedi traditional healers in the Limpopo Province | | | | | | |
| --- | --- | --- | --- | --- | --- | --- |
| **Scientific name**  **(Voucher no.)** | **Botanical family** | **Vernacular name** | **Part/s used** | **Mode of preparation and administration** | **Ailment/s or health-related problems treated** | **Frequency**  **(%)** |
| 1. *Acacia senegal*   var. *Kerensis*  (SS102) | Fabaceae | Mookanathutlwa | Seed | Pounded and 5 teaspoons taken with a tin cup of warm water. Thrice a day | Diarrhoea | 14.7 |
|  |  |  | Seed & bark | Mixed and burned thrice a day; smoke inhaled | Nose bleeding | 2.9 |
| 1. *Acanthus montanus* L   ( SS 76) | Acanthaceae | Unknown | Root | Boiled for 20 minutes and one tin cup of extract is taken orally. Thrice a day | Stomach complain | 2.9 |
| 1. *Aframomum melegueta* (Rox.) K.Schum.   (SS 331) | Zingiberaceae | Unknown | Root | Boiled for 10 minutes and one tin cup of extract is taken orally. Thrice a day | Tuberculosis | 2.9 |
| 1. *Agapanthus inarpetus* P. Beauv. subsp. *inarpetus*   (SS 340) | Agapanthaceae | Leta-la-phofu | Root | Boiled for 10 minutes and one tin cup of extract is taken orally. Thrice a day | Tuberculosis | 2.9 |
| 1. *Agave americana* L.   (SS 02) | Agavaceae | Mobepi | Leaf | Boiled for 25 minutes and one tin cup of extract is taken orally. Thrice a day | Hypertension | 2.9 |
| 1. *Aloe aborescens* Mill.   (SS 59) | Asphodelaceae | Kgopha-ya-fase | Root | Boiled for 20 minutes and one tin cup of extract is taken orally. Thrice a day | HIV/AIDS | 5.7 |
| 1. *Aloe falcata* Baker   (SS 330) | Asphodelaceae | Kgopha | Leaf | Boiled for 5 minutes and one tin cup of extract is taken orally. Thrice a day | Hypertension | 2.9 |
| 1. *Aloe angolensis* Baker   (SS 42) | Asphodelaceae | Sekgophane | Leaf | Boiled for 25 minutes and one tin cup of extract is taken orally. Thrice a day | Appetite | 2.9 |
| 1. *Aloe marlothii* A. Berger subsp. *marlothii*   (SS 80) | Asphodelaceae | Kgopha-ya-go-ema | Leaf & root | Mixed and boiled for 5–20 minutes and one tin cup of extract is taken orally. Thrice a day | Diabetes mellitus and Chlamydia | 8.8 |
|  |  |  | Root | Boiled for 10 minutes and one tin cup of extract is taken orally. Thrice a day | Gonorrhoea | 8.8 |
|  |  |  | Leaf | Boiled for 10 and one tin cup of warm extract is administered anally by a healer via bulb syringe. Once daily for a week | Chlamydia | 2.9 |
| 1. *Alternanthera pungens* Kunth   (SS 402) | Amaranthaceae | Mosweetswe | Tuber | Chopped and macerated in cow’s milk for 24 hours. Decoction is taken orally. Trice a day | Gonorrhoea | 2.9 |
| 1. *Ammocharis coranica* (Ker Gawl.) Herb.   (SS 201) | Amaryllidaceae | Unknown | Root | Boiled for 20 minutes and one tin cup of extract is taken orally. Thrice a day | Erectile dysfunction | 2.9 |
| 1. *Artemisia afra* Jacq. ex Willd. var. afra   ( SS 223) | Asteraceae | Lengana | Leaf | Crushed and smoked with a newspaper. Twice a day | Tuberculosis | 5.8 |
|  |  |  |  | Boiled for 15 minutes and one tin cup of extract is taken orally. Thrice a day | Tuberculosis | 2.9 |
|  |  |  |  | Crushed and mixed with *Mentha* spp (crushed leaves) and smoked with a newspaper. Thrice a day | Tuberculosis | 5.8 |
|  |  |  |  | Burned in a hut thrice a day; smoke is inhaled | Tuberculosis | 5.8 |
|  |  |  |  | Deposited in the hot water thrice a day; steam is inhaled | Tuberculosis | 5.8 |
| 1. *Artemisia annua* L.   (SS 43) | Asteraceae | Mohlaswapatla | Root | Boiled for 20 minutes and one tin cup of extract is taken orally | Erectile dysfunction | 2.9 |
| 1. *Asparagus* *falcatus* L.   (SS 332) | Asparagaceae | Mophatlalatsa-maru | Root | Mixed with *Aloe aborescens* (root), *Elephantorrhiza elephantina* (root). Boiled for 5 minutes. One tin cup of extract is taken orally. Thrice a day | Blood clotting | 2.9 |
| 1. *Bidens pilosa* L.   (SS 214) | Asteraceae | Mophodisa/ mokolonyane | Root | Boiled for 15 minutes and one tin cup of extract is taken orally. Thrice a day | Menstrual disorder | 2.9 |
| 1. *Boscia albitrunca* (Burch.) Gilg & Gilg-Ben.   (SS 300) | Capparaceae | Mohlophi | Root | Mixed with *Elephantorrhiza elephantina* (root), *Plectranthus ciliatus* (root), *Peltophorum* *africanum* (root). Boiled for 20 minutes. One tin cup of extract is taken orally. Thrice a day | HIV/AIDS | 2.9 |
| 1. *Brachylaena discolor* DC.   (SS 31) | Asteraceae | Mphahla | Root /bark | Boiled for 20 minutes and one tin cup of extract is taken orally. Four times a day | Female infertility | 5.8 |
| 1. *Burkea africana* Hook.   (SS 60) | Fabaceae | Monatlo | Leaf | Pounded and 5 teaspoons applied topically every time after bathing | Circumcision wounds | 2.9 |
| 1. *Caesalpinia decapetala* (Roth) Alston.   (SS 74) | Fabaceae | Mokgabane | Root | Boiled for 10 minutes and one tin cup of extract is taken orally. Thrice a day | Gonorrhoea | 2.9 |
| 1. *Callilepis laureola* DC.   (SS 52) | Asteraceae | Phela | Tuber | Boiled for 5–10 minutes and a cup of extract is taken orally. Thrice a day | Erectile dysfunction | 2.9 |
|  |  |  |  |  | kidney problem | 5.9 |
|  |  |  |  |  | Low sperm count | 2.9 |
| 1. *Callilepis* *salicifolia* Oliv.   (SS 62) | Asteraceae | Phelana | Tuber | Boiled for 5–20 minutes and one tin cup of extract is taken orally. Thrice a day | Gonorrhoea | 2.9 |
|  |  |  |  |  | HIV/AIDS | 2.9 |
|  |  |  |  |  | Leukemia | 5.8 |
| 1. *Cannabis sativa* L. var. *sativa*   (SS 24) | Cannabaceae | Mopatse | Leaf | Macerated in warm water for 24 hours and one tin cup of decoction is taken orally. Thrice a day | Tuberculosis | 2.9 |
| 1. *Capsicum chinese* L.   (SS 40) | Solanaceae | Mopherefere | Root | Boiled for 15 minutes and one tin cup of extract is taken orally. Thrice a day | Period pains | 2.9 |
| 1. *Carica papaya* L.   (SS 70) | Caricaceae | Mophopho “wapoo” | Root | Pounded and 5 teaspoons taken orally with a bowl of soft porridge. Thrice a day | Erectile dysfunction | 11.7 |
|  |  |  | Fruit | Squeezed juice from unripe fruit and one tin cup of juice is taken orally. Thrice a day | Abortion | 2.9 |
|  |  |  | Root | Boiled for 20 minutes and one tin cup of extract is taken orally. Thrice a day | Diabetes mellitus | 2.9 |
|  |  |  | Root | Mixed with *Cucumis myriocarpus* (tuber). Boiled for 20 minutes. One tin cup of extract is taken orally. Thrice a day | Gonorrhoea | 2.9 |
| 1. *Carissa bispinosa* (L.) Desf. ex Brenan   (SS 104) | Apocynaceae | Motshukudu | Thorn | Boiled for 20 minutes thrice a day; steam is inhaled | Body pains | 2.9 |
| 1. *Carpobrotus edulis* (L.) L. Bolus subsp. *edulis*   (SS 23) | Mesembyanthemaceae | Lepolomo-la-go-naba | Leaf | Squeezed juice and one tin cup of juice is taken orally. Thrice a day | Diabetes mellitus and goiter | 2.9 |
| 1. *Cassia abbreviata* subsp. *abbreviata*   (SS 67) | Fabaceae | Monêpenepe | Root | Boiled for 5 minutes and undisclosed volume of the extract is used to bath | Measles | 8.8 |
| 1. *Catha edulis* (Vahl) Forssk. ex Endl.   (SS 77) | Celastraceae | Lewane | Root | Boiled for 5–10 minutes and one tin cup of extract is taken orally. Thrice a day | Diarrhoea | 2.9 |
|  |  |  |  |  | Erectile dysfunction | 32.4 |
| 1. *Catharanthus roseus* (L.) G. Don   (SS 33) | Apocynaceae | Lepolomo-le-le-pinki-la *drop* | Root | Boiled for 5–20 minutes | Gonorrhoea | 58.8 |
|  |  |  |  | Boiled for 5 minutes and one tin cup of warm extract is administered anally via bulb syringe by a healer. Once | Gonorrhoea | 2.9 |
| 1. *Centella asiatica* (L.) Urb.   (SS 91) | Apiaceae | Unknown | Root | Boiled for 7 minutes and one tin cup of extract is taken orally. Thrice a day | Tonsils | 2.9 |
| 1. *Ceropegia purpurascens* subsp. *purpurascens*   (SS 39) | Apocynaceae | Monamela | Root | Boiled for 10 minutes and one tin cup of extract is taken orally. Thrice a day | Erectile dysfunction | 2.9 |
| 1. *Conyza scabrida* DC.   (SS 10) | Asteraceae | Unknown | Root | Boiled for 5 minutes and one tin cup of extract is taken orally. Thrice a day | Depression | 2.9 |
| 1. *Chamaesyce prostrate* (Aiton) Small   (SS 213) | Euphorbiaceae | Sesese | Whole plant | Boiled for 20 minutes and one tin cup of extract is taken orally. Thrice a day | Womb problem | 2.9 |
| 1. *Chironia baccifera* L.   (SS 22) | Gentianaceae | Mahlo-a-Mmutla | Root | Boiled for 20 minutes and one tin cup of extract is taken orally. Thrice a day | Blood clotting | 2.9 |
|  |  |  |  |  | Tuberculosis | 2.9 |
| 1. *Cinnamomum verum* J. Presl   (SS 337) | Lauraceae | Mokwere-kwere-o- mogolo | Root | Mixed with *Burkia africana* (root), *Hypoxis hemerocallidea* (tuber), *Geigeria aspera* (entire plant). Boiled for 5 minutes. One tin cup of extract is taken orally. Thrice a day | HIV/AIDS | 2.9 |
| 1. *Citrullus lanatus* (Thunb.) Matsum. & Nakai   (SS 09) | Cucurbitaceae | Morotse | Seed | Fried and eaten as snack | Appetite | 2.9 |
| 1. *Citrus lemon* (L.) Burm. F.   (SS 480) | Rutaceae | Moswiri | Root | Mixed with *Acanthus montanus* (root), *Carica papaya* (root), *Zea* *mays* (root) and boiled for 20 minutes. One tin cup of extract is taken orally. Thrice a day | Malaria | 2.9 |
| 1. *Combretum hereroense* subsp. grotei   (SS 440) | Combretaceae | Mokata, and Molepa | Seed | Burned in a hut thrice a day; smoke is inhaled | Tuberculosis | 2.9 |
|  |  |  | Bark | Boiled for 20 minutes and one tin cup of extract is taken orally. Thrice a day | Tuberculosis | 2.9 |
| 1. *Combretum molle* R. Br. ex G. Don   (SS 219) | Combretaceae | Mokgwethe | Root | Pounded and mixed 2 teaspoon of salt and applied topically after bathing | Wound /general injuries | 2.9 |
| 1. *Commelina africana* L.   (SS 96) | Commelinaceae | Kgopo, and bjangmphara | Root | Mixed with *Helichrysum caespititium* (whole plant) and pounded. Five teaspoons is taken orally with a cup of warm water. Thrice a day | Hypertension | 2.9 |
| 1. *Commiphora marlothii* Engl.   (SS 403) | Burseraceae | Mphapha | Bark/root | Pounded and taken orally with warm water. Thrice a day | Diarrhoea | 29.4 |
|  |  |  | Bark | Burned and smoke is inhaled | Epilepsy | 2.9 |
| 1. *Cotyledon orbiculata* L.   (SS 37) | Grassulaceae | Tsebe ya kolobe | Root | Chopped and macerated in warm water for 24 hours. One tin cup of the decoction is taken orally. Thrice a day | Gonorrhoea | 2.9 |
| 1. *Croton pseudopulchellus* Pax   (SS 51) | Euphorbiaceae | Sehlare sa pelo | Leaf | Boiled (undisclosed time) and one tin cup of extract is taken orally. Thrice a day. | Asthma and heart problem | 2.9 |
| 1. *Cussonia paniculata* subsp. *paniculata*   (SS 50) | Araliaceae | Malebathe | Root | Boiled for 5 minutes and 3 teaspoons of salt is added to the extract (2l bottle). One tin cup of the mixture is taken orally. Thrice a day | Stomach complain | 2.9 |
| 1. *Cucumis myriocarpus* subsp. *leptodermis*   (SS 35) | Cucurbitaceae | Magapyana | Root | Boiled for 20 minutes and one tin cup of extract is taken orally. Thrice a day | Chlamydia | 2.9 |
| 1. *Cussonia spicata* Thunb.   (SS 75) | Araliaceae | Motšhetšhe | Root | Boiled for 5–10 minutes and one tin cup of extract is taken orally. Thrice a day | Appetite and diabetes mellitus | 2.9 |
| 1. *Cyperus papyrus* L.   (SS 97) | Cyperaceae | Mohlaka | Whole plant | Boiled for 10–30 minutes and one tin cup of extract is taken orally. Thrice a day | Menstrual disorder | 2.9 |
| 1. *Datura stramonium* L.   (SS 41) | Solanaceae | Lechoe/thoba | Seed | Pounded and mixed with Vaseline in a container (500g). Five teaspoons of mixture is applied topically to massage affected area. Thrice a day | Stroke | 2.9 |
| 1. *Dicoma anomala* subsp. *anomala*   (SS 407) | Asteraceae | Hlonya | Tuber | Macerated in warm water for 24 hrs and one tin cup of the decoction is taken orally. Thrice a day | Diarrhoea | 2.9 |
|  |  |  |  | Boiled for 5–25 minutes and one tin cup of extract is taken orally. Thrice a day | Contaminated blood (STIs) and measles | 2.9 |
|  |  |  |  | Pounded and 3 teaspoons is applied on incision made by a healer with a razor blade. Thrice a day | Swelled leg | 2.9 |
| 1. *Dioscorea sylvatica* var. brevipes.   (SS 11) | Dioscoreaceae | Monamela | Root | Mixed with *Aloe marlothii* (root) and boiled for 20 minutes. One tin cup of extract is taken orally. Thrice a day | Gonorrhoea | 2.9 |
| 1. *Dodonaea viscose* var. *angustifolia*   (SS 117) | Sapindaceae | Mofenshe | Root | Mixed with *Citrullus lanatus* (root), *Euclea crispa* (root) and boiled for 20 minutes. One cup of extract is taken orally. Thrice a day | HIV/AIDS | 2.9 |
| 1. *Dolichos falciformis* E. Mey.   (SS 36) | Fabaceae | Sepeiti | Root | Macerated in warm water for 24 hrs and one tin cup of warm decoction is administered by healer anally via bulb syringe. Twice a week | Blood purifier | 2.9 |
| 1. *Dombeya rotundifolia* var. *rotundifolia*   (SS 301) | Malvaceae | Mohlabaphala | Root | Boiled for 5–20 minutes and one tin cup of extract is taken orally. Thrice a day | Diarrhoea and hypertension | 32.5 |
| 1. *Drimia elata* Jacq.   (SS 18) | Hyacinthaceae | Sekanama | Bulb | Boiled for 5–20 minutes and one tin cup of extract is taken orally. Thrice a day | Blood purifier | 8.8 |
|  |  |  |  |  | Female infertility | 5.8 |
|  |  |  |  |  | Gonorrhoea and | 2.9 |
|  |  |  |  |  | Hypertension | 8.8 |
| 1. *Elaeodendron transvaalense* (Burtt Davy) Rott. Archer   (SS 32) | Celastraceae | Monamane | Root/bark | Mixed with *Peltophorum africanum* (bark) and boiled for 20 minutes. One tin cup of extract is taken orally. Thrice a day | Female infertility | 2.9 |
| 1. *Elephantorrhiza elephantina* (Burch.) Skeels   (SS 100) | Fabaceae | Mosehlana/ moshisane | Root | Mixed with *Peltophorum* *africanum* (bark). Boiled for 20 minutes and one tin cup of extract is taken orally. Thrice a day | HIV/AIDS | 2.9 |
|  |  |  |  | Boiled for 5–20 minutes and one tin cup of extract is taken orally. Thrice a day | Blood clotting | 8.8 |
|  |  |  |  |  | Blood purifier | 14.7 |
| 1. *Equisetum ramosissimum* Def, subsp. *ramosissimum* L.   (SS 49) | Equisetaceae | Unknown | Root | Boiled for 5 minutes and one tin cup of extract is taken orally. Thrice a day | Asthma | 2.9 |
| 1. *Encephalartos transvenosus* Stapf & Burtt Davy   (SS 63) | Zamiaceae | Mofaka | Root | Macerated in water for 24 hours and one tin cup of the decoction is taken orally. Thrice a day. | Hypertension | 2.9 |
| 1. *Englerophytum magalismontanum* (Sond.) T.D. Penn.   (SS 48) | Sapotaceae | Mohlatswa | Bark | Boiled for 5–20 minutes and one tin cup of extract is taken orally. Thrice a day | Diabetes mellitus | 5.8 |
| 1. *Eriobotrya japonica* (Thunb.) Lindl.   (SS 311) | Rosaceae | Unknown | Leaf | Boiled for 7–10 minutes and one tin cup of extract is taken orally. Thrice a day | Hypertension and  tuberculosis | 2.9 |
| 1. *Eucalyptus camaldulensi*s Dehnh.   (SS 401) | Myrtaceae | Mopilikomo | Leaf | Boiled for 5–20 minutes and one tin cup of extract is taken orally. Thrice a day | Tuberculosis | 2.9 |
| 1. *Encephalartos woodii* Sander   (SS 71) | Zamiaceae | Senamane | Bark | Boiled for 30 minutes and one tin cup of extract is taken orally. Eight times a day | Mental illness | 2.9 |
| 1. *Euclea crispa* subsp. *crispa*   (SS 57) | Ebenaceae | Mokwerekwere | Root | Mixed with *Aloe falcata* (root) and boiled or 20 minutes. One tin cup of extract is taken orally. Thrice a day | HIV/AIDS | 2.9 |
| 1. *Eucomis pallidiflora* subsp. *pole*-*evansii*   (SS 355) | Hyacinthaceae | Mathuba-difala | Bulb | Boiled for 5–8 minutes and one tin cup of extract is taken orally. Thrice a day | Tuberculosis | 8.8 |
|  |  |  |  | Boiled for 5–25 minutes and one tin cup of extract is taken orally thrice a day | Blood clotting | 14.5 |
|  |  |  |  |  | Blood purifier | 8.8 |
|  |  |  |  | Boiled (undisclosed time) and one tin cup of extract is taken orally. Thrice a day | Tuberculosis | 2.9 |
|  |  |  |  | Mixed with *Ziziphus mucronata* (root) and boiled for 20 minutes. One tin cup of extract taken orally three thrice a day | Chlamydia | 2.9 |
| 1. *Euphorbia ingens* E. Mey. ex. Boiss.   (SS 34) | Euphorbiaceae | Mohlohlokgomo | Stem | Applied topically (raw prescription) directly in to the incision on breast made by a healer, every time after bathing. | Breast cancer | 8.8 |
| 1. *Euphorbia maleolens* E. Phillips   (SS 226) | Euphorbiaceae | Rofa-bja-Tau | Whole plant | Mixed with *Triumfetta* sp. (root), Z*anthoxylum humile* (root); pounded and 5 teaspoons taken orally with a bowl of soft porridge. Thrice a day | HI/AIDS | 2.9 |
|  |  |  |  | Boiled for 20 minutes and one tin cup of extract is taken orally. Thrice a day | HIV/AIDS | 2.9 |
|  |  |  |  | Mixed with *Myrothamnus flabellifolius* (entire plant); pounded and 5 teaspoons taken orally with a bowl of soft porridge. Thrice a day | HIV/AIDS | 2.9 |
|  |  |  |  | Mixed with *Triumfetta* sp. (root) and *Zanthoxylum humile* (root); pounded and 5 teaspoons taken orally with a bowl of soft porridge. Thrice a day | HIV/AIDS | 2.9 |
| 1. *Ficus carica* L. subsp. rupestris (Hausskn.) Browicz (Dncir)   (SS 89) | Moraceae | Mofeiye | Bark | Boiled for 10 minutes and one tin cup of extract is taken orally. Thrice a day | Tuberculosis | 2.9 |
| 1. *Ficus platypoda* A. Cunn. ex Miq.   (SS 323) | Moraceae | Unknown | Root | Boiled for 20 minutes and one tin cup of extract is taken orally. Thrice a day | Tuberculosis | 2.9 |
| 1. *Ficus sycomorus* L. subsp. *gnaphalocarpa* (Mig.) C.C Berg   (SS 56) | Moraceae | Mothekese/ Mohlono/mogobagoba | Bark and root | Boiled for 6 minutes and one tin cup of extract taken orally. Thrice a day | Goiter | 2.9 |
| 1. *Geigeria aspera* Harv. var*. aspera*   (SS 310) | Asteraceae | Makgonatsohle | Whole plant | Burned in a hut thrice a day; smoke is inhaled | Nose bleeding | 2.9 |
| 1. *Gethyllis namaquensis* (Schonland) Oberm.   (SS 83) | Amaryllidaceae | Naka tsa tholo | Bulb | Chopped and macerated in warm water for 24 hours and one tin cup of extract is taken orally. Thrice a day | Chlamydia | 2.9 |
|  |  |  |  | Boiled for 10 minutes and one tin cup of extract is taken orally. Thrice a day | Diabetes mellitus |  |
| 1. *Gomphocarpus fruticosus* subsp. *fruticosus*   (SS 101) | Apocynaceae | Mosotsa poo | Root | Boiled for 15 minutes and one tin cup of extract is taken orally. Thrice a day | Erectile dysfunction | 14.5 |
| 1. *Grewia bicolor* Juss. var. *bicolor*   (SS 16) | Malvaceae | Mothetlwa | Root | Boiled for 5–30 minutes and one tin cup of extract is taken orally. Thrice a day | Diarrhoea | 47 |
| 1. *Gymnosporia senegalensis* (Lam.) Loes.   (SS 79) | Celastraceae | Mophato | Root/ leaf | Boiled for 5–20 minutes and one tin cup of extract is taken orally. Thrice a day. | Blood clotting | 5.8 |
|  |  |  |  |  | Diarrhoea | 8.8 |
|  |  |  |  |  | Erectile dysfunction | 14.5 |
| 1. *Gymnosporia tenuispina* (Sond.) Szyszyl.   (SS 333) | Celastraceae | Sepeiti | Root | Boiled for 20 minutes and drops of extract is applied via a finger on the eyes whenever the pain is felt | Eye infection | 2.9 |
| 1. *Helichrysum caespititium* (DC.) Harv.   (SS 78) | Asteraceae | Bokgatha/Mabjana/Mmeetse | Whole plant | Boiled for 10–20 minutes or pounded and taken orally with a tin cup with warm water or a bowl of soft porridge. Thrice a day | Diabetes mellitus | 5.8 |
|  |  |  |  | Burned a smoke is inhaled | Epilepsy | 2.9 |
|  |  |  |  | Boiled for 20 minutes and one tin cup of the extract is orally taken. Thrice a day | Hypertension | 8.8 |
|  |  |  |  |  | Blood purifier | 8.8 |
|  |  |  |  | Boiled for 10 minutes and one tin cup of extract is taken orally | Measles | 8.8 |
|  |  |  |  | Boiled for 20 minutes and one tin cup of extract is taken orally | Gonorrhoea | 2.9 |
|  |  |  |  | Boiled for 5–20 minutes and one tin cup of extract is taken orally. Thrice a day | Diarrhoea | 8.8 |
| 1. *Helichrysum herbaceum* (Andrews) Sweet   (SS 207) | Asteraceae | Mohlomela-tsie | Root | Boiled for 20 minutes and one tin cup of extract is taken orally. Thrice a day | Heart problem | 2.9 |
| 1. *Hermannia quartiniana* A. Rich.   (SS 107) | Malvaceae | Unknown | Root | Boiled for 20 minutes and one tin cup of extract is taken orally. Thrice a day | Diabetes mellitus | 2.9 |
| 1. *Hypoxis hemerocallidea* (Fisch.) Mey. & Avé–Lall   (SS 115) | Hypoxidaceae | Titikwane/ sesogadi | Tuber | Mixed with *Senna italica* (root) and pounded; 5 teaspoons taken orally with a tin cup of warm water. Thrice a day | Gonorrhoea | 8.8 |
|  |  |  |  | Pounded and 5 teaspoons taken orally with a bowl of soft porridge. Thrice a day | HIV/AIDS | 2.9 |
|  |  |  |  | Boiled for 20 minutes and one tin cup of extract is taken orally. Thrice a day | Gonorrhoea | 2.9 |
|  |  |  |  | Boiled for 10 minutes and one tin cup of extract is taken orally. Thrice a day | Tuberculosis | 2.9 |
| 1. *Hypoxis iridifolia* Baker   (SS 68) | Hypoxidaceae | Monna maledu, modiboya | Root | Boiled for 5–10 minutes. One tin cup of extract is taken orally. Thrice a day | Diabetes mellitus | 11.4 |
| 1. *Hypoxis obtusa* Burch. ex Ker Gawl.   (SS 336) | Hypoxidaceae | Monna maledu/ Swikiri poo | Tuber | Mixed with *Ziziphus mucronata* (root) and pounded. Five teaspoons taken with a cup of warm water | Chlamydia | 2.9 |
|  |  |  |  | Boiled for 20 minutes and one tin cup of warm extract is administered by healer anally via fatal bulb syringe. Thrice week | Blood clotting | 2.9 |
|  |  |  |  | Boiled for 20 minutes and one tin cup of extract is taken orally. Thrice a day | Contaminated blood | 2.9 |
|  |  |  |  | Pounded and 5 teaspoons is administered by healer anally via fatal bulb syringe. Once a day | Contaminated blood | 2.9 |
|  |  |  |  | Mixed with *Dombeya roundifolia* (root) and boiled for 5 minutes. One tin cup of extract is taken orally. Thrice a day | Hypertension | 2.9 |
|  |  |  |  | Mixed with *Eucomis* *pallidiflora* (bulb) and pounded. One tin cup of the extract is administered by healer anally via fatal bulb syringe. Thrice a week | Erectile dysfunction | 11.4 |
| 1. *Ipomoea obscura* var. *obscura*   (SS 200) | Convolvulaceae | Kgomodimaswi | Root | Boiled for 20 minutes and one tin cup of extract is taken orally. Thrice a day | Gonorrhoea | 2.9 |
| 1. *Jatropha curcas* L.   (SS 120) | Euphorbiaceae | Sehlare sa banna | Root | Boiled for 5 minutes and one tin cup of extract is taken orally. Thrice a day | Erectile dysfunction | 2.9 |
| 1. *Justicia petiolaris* subsp. bowiei   (SS 06) | Acanthaceae | Unknown | Root | Boiled for three minutes and a cup of extract is taken orally. Thrice a day | Stomach complain | 2.9 |
| 1. *Kirkia wilmsii* Engl.   (SS 94) | Kirkiaceae | Legaba/modumela | Tuber | Five to ten piled tubers is eaten as raw after every meals. Thrice a day | Hypertension | 94.1 |
|  |  |  |  | Pounded and taken orally with a warm tin cup of water. Thrice a day | Hypertension | 2.9 |
| 1. *Kleinia longiflora* DC.   (SS 217) | Asteraceae | Lekgabolo/ motlalamaswi | Whole plant | Boiled for 20 minutes and one tin cup of extract is taken orally.. Twice a day | Sepsis | 5.8 |
|  |  |  | Root | Boiled for 20 minutes and one tin cup of extract is taken orally. Twice a day | Chlamydia | 2.9 |
|  |  |  |  | Macerated in warm water for 24 hrs and one tin cup of the decoction is administered by healer anally via bulb syringe. Twice week | Infertility in woman | 2.9 |
| 1. *Lantana camara* L.   (SS 324) | Verbenaceae | Sebabane | Root | Boiled for 5 minutes and one tin cup of extract taken orally. Thrice a day | Hypertension | 5.8 |
| 1. *Lessertia microphylla* (Burch. ex DC.) Goldblatt & J.C. Manning   (SS 93) | Fabaceae | Mosapelo | Root | Boiled for 10 minutes and one tin cup of extract is taken orally. Thrice a day | Diabetes mellitus | 5.8 |
| 1. *Leonotis leonurus* (L.) R. Br.   (SS 334) | Lamiaceae | Lebake | Leaf/ stem | Boiled for 10 minutes and one tin cup of extract is taken orally. Thrice a day | Headache | 2.9 |
| 1. *Lippia javanica* (Burm. F.) Spreng.   (SS 180) | Verbenaceae | Mosunkwane | Leaf | Boiled for 5 minutes and one tin cup of extract is taken orally. Thrice a day | Chest complain and tuberculosis | 2.9 |
|  |  |  |  | Burned in a hut thrice a day; smoke inhaled | Nose bleeding and tuberculosis | 2.9 |
|  |  |  |  | Deposited in hot water thrice a day; steam is inhaled | Tuberculosis | 5.8 |
| 1. *Lolium multiflorum* Lam.   (SS 15) | Poaceae | Botsakatsaka | Whole plant | Boiled for 20 minutes and one tin cup of extract is taken orally. Thrice a day | Kidney problem | 2.9 |
| 1. *Medicago sativa* L.   (SS 320) | Fabaceae | Luserene | Whole plant | Mixed with *Croton* *pseudopulchellus* (leaves) and Boiled for 20 minutes. One tin cup of the extract is taken orally. Thrice a day | Heart problem | 2.9 |
| 1. *Melissa officinalis* L.   (SS 03) | Labiatae | Unknown | Leaf | Boiled for 20 minutes and one tin cup of the extract is taken orally. Thrice a day | Hypertension | 1.9 |
| 1. *Mentha* spp   (SS 477) | Lamiaceae | Mominti | Whole plant | Crashed and smoked with a newspaper. Twice a day | Tuberculosis | 2.9 |
| 1. *Merwilla plumbea*  (Lindl.) Speta.   (SS 338) | Hyacinthaceae | Sekakgopha | Leaf | Boiled for 5–10 minutes and one tin cup of extract is taken orally. Thrice a day | Diabetes mellitus | 8.8 |
| 1. *Mimusops zeyheri* Sond.   (SS 53) | Sapotaceae | Mmupudu | Leaf | Boiled for 10–25 minutes and one tin cup of extract is taken orally. Thrice a day | Diabetes mellitus | 14.7 |
| 1. *Momordica balsamina* L.   (SS 99) | Cucurbitaceae | Mothwatwa | Root | Boiled for 5–10 minutes and one tin cup of extract is taken orally. Thrice a day | Diabetes mellitus | 5.8 |
| 1. *Moringa oleifera* Lam.   (SS 65) | Moringaceae | Makgonatsohle | Seed and leaf | Boiled for 5–10 minutes and one tin cup of extract is taken orally. Thrice a day | Diabetes mellitus | 8.8 |
| 1. *Mormordica charantia* L.   (SS 103) | Cucurbitaceae | Monamelala | Leaf | Boiled for 20 minutes and one tin cup of extract is taken orally. Thrice a day | Diabetes mellitus | 2.9 |
| 1. *Mundulea sericea* subsp. *sericea*   (SS 05) | Fabaceae | Mosetla tlou/ motlou | Root | Boiled for 10–30 minutes and one tin cup of extract is taken orally. Thrice a day | Menstrual disorder | 2.9 |
| 1. *Musa sapientum* L.   (SS 307) | Musaceae | Mopanana | Leaf | Mixed with *Hypoxis obtusa* (tuber), scale of an ostrich egg and burned; 3 teaspoons of resulting ashes is applied topically | Wound /general injuries | 2.9 |
| 1. *Myrothamnus flabellifolius*  Welw   (SS 111) | Myrothamnaceae | Boka, fenya, Makgonatsohle/Tsoga | Whole plant | Burned in hut twice a day; smoke is inhaled | Nose bleeding | 5.8 |
|  |  |  |  | Pounded and one tin cup is taken orally with warm water. Thrice a day | Erectile dysfunction | 8.8 |
|  |  |  |  | Boiled for 5–15 minutes one tin cup of the extract is taken orally. Thrice a day | Tuberculosis | 11.4 |
|  |  |  |  | Burned in a hut four times a day; smoke is inhaled | Tuberculosis | 2.9 |
| 1. *Olea europea* subsp. *Africana*   (SS 335) | Oleaceae | Motholoari | Bark | Boiled for 15 minutes and drops of the extract is applied in the ear via a bird’s further. Seven times a day | Ear infection | 8.8 |
| 1. *Opuntia ficus*-*indica* Mill.   (SS 90) | Cactaceae | Motloro | Root | Boiled for 20 minutes and one tin cup of the extract is taken orally. Thrice a day | Hypertension | 5.8 |
|  |  |  |  | Boiled for 20 minutes solely or as a mixture with *Ziziphus mucronata* (root). One tin cup of extract is taken orally. Thrice a day | Gonorrhoea | 2.9 |
| 1. *Osyris lanceolata* Hochst. & Steud.   (SS 61) | Santalaceae | Mphera | Root | Boiled for 5–20 minutes and one tin cup of the extract is taken orally. Thrice a day | Diarrhoea | 14.7 |
|  |  |  |  | Pounded and one tin cup is taken orally with warm water. Thrice a day | Diarrhoea | 2.9 |
|  |  |  |  | Boiled for 10–20 minutes and one tin cup of the extract is taken orally. Thrice a day | Erectile dysfunction | 11.4 |
|  |  |  |  |  | Menstrual disorder | 2.9 |
| 1. *Ozoroa sphaerocarpa* R. Fern. & R. Fern.   (SS 30) | Anacardiaceae | Momoko, and monoko | Bark/root | Boiled for 5–20 minutes and one tin cup of the extract is taken orally. Thrice a day | Diarrhoea | 8.8 |
|  |  |  |  |  | Measles | 2.9 |
|  |  |  |  | Pounded and taken orally with a bowl of soft porridge. Thrice a day | Erectile dysfunction | 25.5 |
| 1. *Pappea capensis* Eckl. & Zeyh.   (SS 114) | Sapindaceae | Mongatane | Fruit | Taken as raw. (undisclosed dosage) | Diarrhoea | 8.8 |
| 1. *Pelargonium* spp   (SS 04) | Geraniaceae | Selumi | Root | Boiled for 5–20 minutes and one tin cup of the extract is taken orally. Thrice a day | Female infertility | 8.8 |
|  |  |  |  | Boiled for 20 minutes and undisclosed volume is taken orally. Thrice daily | HIV/AIDS | 2.9 |
|  |  |  |  | Pounded and six table spoons taken orally. Thrice daily with either warm water or porridge | HIV/AIDS | 2.9 |
|  |  |  |  | Boiled for 10–20 minutes and one tin cup the extract is taken orally. Four times a day | Hypertension | 8.8 |
| 1. *Pellaea calomelanos* (Sw.) Link. var. *calomelanos*   (SS 25) | Sinopteridaceae | Lehorometso | Root | Boiled for 15 minutes and one tin cup of extract is taken orally. Thrice a day | Tuberculosis | 2.9 |
| 1. *Peltophorum africanum* Sond.   (SS 13) | Fabaceae | Mosehla | Bark | Mixed with *Elephantorrhiza elephantina* (root) and boiled for 20 minutes. One tin cup of extract is taken orally. Thrice a day | HIV/AIDS | 2.9 |
|  |  |  |  | Pounded and one tin cup is taken orally with warm water. Thrice a day | Erectile dysfunction | 11.4 |
|  |  |  |  |  | Post partum |  |
|  |  |  |  | After being boiled for 15 minutes, 10 teaspoons of FG and Joko teas are added to the extract (2 L bottle). One tin cup of the mixture is taken orally. Thrice a day | Female infertility | 2.9 |
|  |  |  |  | Boiled for 5–20 minutes and one tin cup of extract is taken orally. Thrice a day | Female infertility | 2.9 |
| 1. *Persea americana* Mill.   (SS 92) | Lauraceae | Moafokhathe | Root | Boiled for 10–20 minutes and one tin cup of extract is taken orally. Thrice a day | Diabetes mellitus | 2.9 |
|  |  |  |  |  | Hypertension | 8.8 |
| 1. *Phytolacca dodecandra* L'Hérit.   (SS 20) | Phytolaccaceae | Mopampara | Root | Boiled for 20–35 minutes and one tin cup of the extract is taken orally. Thrice a day | Female infertility and  menstrual disorder | 2.9 |
| 1. *Plectranthus ciliatus* E. Mey. ex. Benth.   (SS 322) | Lamiaceae | Sehlare sa pelo | Root | Boiled for 5–20 minutes and one tin cup of extract is taken orally. Thrice a day | Heart problem | 2.9 |
| 1. *Plumeria obtusa* L.   (SS 95) | Apocynaceae | Mohlare wa maswi wa sukiri | Leaf | Boiled for 5–10 minutes and one tin cup of the extract is taken orally. Thrice a day | Diabetes mellitus | 8.8 |
| 1. *Polygala* *hottentotta* C. Presl   (SS 17) | Polygalaceae | Lehlokwa la tsela | Whole plant | Boiled for 10 minutes and nine tin cups of the extract is taken orally. Once a day | Constipation | 5.8 |
| 1. *Petroselenium crispum* ([Mill.](http://en.wikipedia.org/wiki/Philip_Miller)) [Fuss](http://en.wikipedia.org/w/index.php?title=Johann_Mih%C3%A1ly_Fuss&action=edit&redlink=1)   (SS 86) | Apiaceae | Morogane | Leaf | Boiled for 5 minutes and one tin cup of the extract is taken orally. Thrice a day | Clean Kidney | 5.8 |
| 1. *Protea caffra* subsp. caffra   (SS 341) | Proteaceae | Unknown | Seed | Pounded and 6 teaspoons are taken orally with warm water | Chlamydia | 2.9 |
| 1. *Prunus persica* (L.) Batsch var. *persica*   (SS 84) | Rosaceae | Moperekisi | Root | Pounded and 5 teaspoons are taken orally with a bowl of soft porridge. Thrice a day | Erectile dysfunction | 8.8 |
| 1. *Psidium guajava* L.   (SS 408) | Myrtaceae | Mokwaba | Root | Mixed with *Punica granatum* (root) and boiled for 5 minutes. One tin cup of the extract is taken orally. Thrice a day | Diarrhoea | 2.9 |
|  |  |  |  | Boiled for 5–10 minutes and one tin cup of the extract is taken orally. Thrice a day | Diarrhoea | 8.8 |
|  |  |  |  | Boiled for 7 minutes and one tin cup of extract is taken orally. Thrice a day | Hypertension | 2.9 |
| 1. *Punica granatum* L.   (SS 73) | Punicaceae | Mokgarenate | Root/pericarp | Boiled for 5–25 minutes and one tin cup of extract is taken orally. Thrice a day | Diarrhoea | 58.8 |
|  |  |  | Root | Boiled for 10–20 minutes and one tin cup of extract is taken orally. Thrice a day | Blood vomiting | 5.8 |
|  |  |  |  |  | Diabetes mellitus | 2.9 |
| 1. *Rhus chinensis* Mill.   (SS 108) | Anacardiaceae | Mohlwehlwe/Mokasebati | Root | Boiled for 5–7 minutes and 3–5 tin cups of extracts are taken orally. Thrice a day | Sepsis | 11.4 |
| 1. *Ricinus communis* var. *communis*   (SS 38) | Euphorbiaceae | Mothoba | Whole plant | Boiled for 5–30 minutes and 1–3 tin cups of warm extract is used to massage affected legs | Swelled leg | 20.5 |
| 1. *Saccharum officinarum* L.   (SS 404) | Poaceae | Momoba | Leaf | Boiled for 20 minutes and one tin cup of the extract is taken orally. Thrice a day | Diarrhoea | 2.9 |
| 1. *Salix mucronata* subsp. *capensis*   (SS 21) | Salicaceae | Mmilo | Fruit | Six-10 fruits are taken orally as raw. Four times a day | Chest complain | 2.9 |
|  |  |  | Seed and fruit | Pounded and 5 tablespoons are taken orally with a tin cup of warm water. Thrice a day | Tuberculosis | 5.8 |
| 1. *Sansevieria hyacinthoides* (L.) Druce   (SS 199) | Dracaenaceae | Makgotse | Leaf | Boiled for 5–10 minutes and one tin cup of the extract is taken orally. Thrice a day | Diarrhoea | 8.8 |
|  |  |  | Root | Boiled for 20 minutes and one tin cup of the extract is taken orally. Thrice a day | HIV/AIDS | 4.9 |
| 1. *Sarcostemma viminale* subsp. *orangeanum*   (SS 106) | Apocynaceae | Mokwere-kwere- o- mogolo | Root/bark | Burned in a hut; smoke is inhaled. Thrice a day | Epilepsy | 2.9 |
|  |  |  | Twig | Boiled for 20 minutes and one tin cup of the extract is taken orally. Thrice a day | Hypertension | 2.9 |
| 1. Schkuhria pinnata (Lam.) Kuntze ex Thell.   (SS 87) | Asteraceae | Sebabane | Whole plant | Boiled for 10–20 minutes and a cup of the extract is taken orally | Hypertension | 5.8 |
|  |  |  |  |  | Blood purifier | 2.9 |
| 1. *Sclerocarya birrea* sub sp. *birrea*   (SS 01) | Anacardiaceae | Morula | Bark | Pounded and taken orally with warm water. Thrice a day | Diarrhoea | 5.8 |
|  |  |  |  | Boiled for 10 minutes and one tin cup of extract is taken orally. Thrice a day | Female infertility | 2.9 |
|  |  |  |  |  | Leukemia | 2.9 |
|  |  |  |  |  | Stroke | 8.8 |
|  |  |  |  | Mixed with *Drimia elata* (bulb), *Elephantorrhiza elephantina* (root), *Elaeodendron transvaalense* (root), *Zanthoxylum capense* (root) and *Sarcostemma viminale* (twigs) and boiled for 10 minutes. One tin cup of the extract is taken orally. Thrice a day | HIV/AIDS | 2.9 |
|  |  |  |  | Mixed with *Drimia elata*, *Eucomis* *pallidiflora* (bulbs) and boiled for 5 minutes. One tin cup of the extract is taken orally. Thrice a day | Blood clotting | 2.9 |
| 1. *Searsia lancea* (L.F.) F.A. Barkley   (SS 227) | Anacardiaceae | Mokalabata/ Motshakhutshakhu | Root | Boiled for 20 minutes and a cup of extract is taken orally | Nta (Bapedi-terminology) | 2.9 |
| 1. *Securidaca longepedunculata* Fresen. var. *longepedunculata*   (SS 07) | Polygalaceae | Mopesu | Root | Mixed with *Zanthoxylum humile* (root) and pounded; 7 teaspoons taken orally with a bowl of soft porridge. Thrice a day | Erectile dysfunction | 2.9 |
| 1. *Senecio barbertonicus* Klatt   (SS113) | Asteraceae | Sehlare sa sejetso | Leaf | Boiled for 20–30 minutes and one tin cup of the extract is taken orally. Thrice a day | Sepsis | 5.8 |
| 1. *Senna didymobotrya* (Fresen.) H.S. Irwin & Barneby   (SS 105) | Fabaceae | Mothekele | Leaf | Boiled for 10 minutes and one tin cup of the extract taken orally. Thrice a day | Blood clotting | 2.9 |
| 1. *Senna italica* subsp. *arachoides*   (SS 321) | Fabaceae | Mankgane/sebetsane | Root | Boiled for 20 minutes and one tin cup of extract is taken orally. Thrice a day | Gonorrhoea | 2.9 |
| 1. *Sesbania punicea* (Cav.) Benth.   (SS 82) | Fabaceae | Mokgabane | Root | Boiled for 20 minutes and one tin cup of extract is taken orally. Thrice a day | Menstrual disorder | 2.9 |
| 1. *Sida cordifolia* subsp. *cordifolia*   (SS 326) | Malvaceae | Sebenyu | Root | Boiled for 10 minutes and cool extract is used for bathing or one tin cup of this extract is taken orally. Thrice a day | Measles | 2.9 |
| 1. *Silver arctotis* (Eng.)   (SS 14) | Asteraceae | Unknown | Whole plant | Pounded and one tin cup of the extract is taken orally with cup of warm water. Thrice a day | Clean kidneys | 2.9 |
| 1. *Siphonochilus aethiopicus* (Schweif.) B.L. Burtt   (SS 202) | Zingiberaceae | Serakulu | Rhizome | Boiled for 5 minutes; steam is inhaled | Asthma | 2.9 |
| 1. *Solanum lycopersicum* L.   (SS 490) | Solanaceae | Motamati | Fruit | Juice is squeezed and 1-3 tin cups of juice is taken orally. Thrice a day | Diarrhoea | 8.8 |
| 1. *Solanum mauritianum* Scop.   (SS 116) | Solanaceae | Mothola-o-mo- tala | Fruit | Macerated in the water for 24 hrs and one tin cup of decoction is administered by healer anally via bulb syringe. Thrice week | Clean kidneys | 2.9 |
| 1. *Solanum panduriforme* E. Mey.   (SS 85) | Solanaceae | Mothola-ye- serolwane | Fruit | Chopped and macerated in warm water for 24 hours. One tin cup of the decoction is taken orally. Thrice a day | Gonorrhoea | 2.9 |
| 1. *Syzygium cordatum* Hochst. ex C. Kranss subsp. *cordatum*   (SS 225) | Myrtaceae | Mohlo | Root and leaf | Boiled for 20 minutes and one tin cup of the extract is taken orally. Every time before and after meals | Ulcer | 2.9 |
| 1. *Tarchonanthus camphoratus* L.   (SS 72) | Asteraceae | Mogata | Root | Boiled for 10–20 minutes and one tin cup of extract is taken orally. Thrice a day | Diabetes mellitus | 2.9 |
|  |  |  |  |  | Hypertension | 5.8 |
| 1. *Terminalia sericea* Burch. ex DC   (SS 112) | Combretaceae | Mogonono | Leaf | Boiled for 5–26 minutes and one tin cup of the extract is taken orally. Thrice a day | Measles | 8.8 |
|  |  |  |  | Pounded and mixed with either olive oil, or Vaseline and used as a lotion after bathing | Measles | 14.7 |
| 1. *Tribulus terestris* L.   (SS 409) | Zygophyllaceae | Mosehlo | Whole plant | Mixed with *Ziziphus mucronata* (root) and boiled for 20 minutes. One tin cup of extract is taken orally. Thrice a day | Chlamydia | 2.8 |
| 1. *Triumffeta* spp   (SS 64) | Tilliaceae | Unknown | Root | Boiled for 5–20 minutes and one tin cup of the extract is taken orally. Thrice a day | Diarrhoea | 5.8 |
|  |  |  |  | Mixed with equal parts of *Euphorbia maleolens* (entire plant), and *Zanthoxylum humile* (root) and pounded; 5 teaspoons taken orally with a bowl of soft porridge. Thrice a day | HIV/AIDS | 2.8 |
| 1. *Turraea obtusifolia* Hochst.   (SS 88) | Meliaceae | Sepeite | Leaf | Mixed with *Triumffeta* sp (root), *Elephantorrhiza elephantina* (root), *Peltophorum africunam* (bark) and boiled for 17 minutes. One tin cup of the extract is taken orally. Thrice a day | Blood purifier | 2.8 |
| 1. *Vigna unguiculata* subsp. *dekindtiana* var. *dekindtiana*   (SS 400) | Fabaceae | Monawa | Leaf | Boiled for 5–20 minutes and one tin cup of the extract is taken orally. Thrice a day | Diarrhoea | 5.8 |
| 1. *Warburgia salutaris* (G. Bertol.) Choir.   (SS 81) | Canellaceae | Molaka | Leaf | Boiled for 5–10 minutes and one or two tin cups of the extract is taken orally. Thrice a day | Cold, flue and measles | 11.4 |
| 1. *Ximenia americana* var. *America*   (SS 460) | Olacaceae | Ikgolotsane | Root | Boiled for 5–10 minutes and one tin cup of the extract is taken orally. Thrice a day | Contaminated blood | 5.8 |
|  |  |  |  |  | Diarrhoea | 11.4 |
| 1. *Zantedeschia aethiopica* (L.) Spreng.   (SS 110) | Araceae | Mothebe | Root | Boiled for 7 minutes and drops of the extract is applied via a stick on infected eyes. Five times a day | Eye infection | 2.9 |
| 1. *Zanthoxylum capense* (Thunb.) Harv.   (SS 511) | Rutaceae | Senokomaropa | Leaf | Burned in a hut; smoke is inhaled. Twice a day | Nose bleeding and tuberculosis | 2.9 |
| 1. *Zanthoxylum humile* (E.A.Bruce) P.G. Waterman   (SS 19) | Rutaceae | Monokwane | Root | Pounded and 5–7 teaspoons is taken orally with a bowl of soft porridge or warm water. Thrice a day | Erectile dysfunction | 32.3 |
|  |  |  |  | Boiled for 5 minutes and one tin cup of extract is taken orally. Thrice a day | Measles | 2.9 |
|  |  |  |  | Mixed with *Euphorbia maleolens* (entire plant), *Triumfetta sp.* (root), *Zanthoxylum humile* (root) and pounded; 5 teaspoons is taken orally with a tin cup of warm water. Thrice a day. | HIV/AIDS | 2.9 |
| 1. *Zea mays* L.   (SS 119) | Poaceae | Mabele | Root | Mixed with *Citrus limon* (leaf) and boiled for 20 minutes. One tin cup of the extract is taken orally. Thrice a day | Malaria | 1.9 |
|  |  |  | Whole plant | Pounded and mixed with Vaseline (lotion) and applied topically every time after bathing | Malaria | 5.8 |
| 1. *Ziziphus mucronata* Wild.   (SS 12) | Rhamnaceae | Mokgalo | Root | Mixed with *Hypoxis obtusa* (tuber), *Ziziphus mucronata* (root) and pounded; 5 teaspoons is taken orally with a tin cup of warm water. Thrice a day | Chlamydia | 2.9 |
|  |  |  |  | Mixed with *Ziziphus mucronata* (root), *Eucomis pallidiflora* (bulb) and pounded; 5 teaspoons taken orally with a tin cup of warm water. Thrice a day | Chlamydia | 5.8 |
|  |  |  |  | Boiled for 20 minutes and one tin cup of extract is taken orally. Thrice a day | Gonorrhoea | 2.9 |
| **KEY**: HIV/AIDS: Human immunodeficiency virus/ acquired immunodeficiency syndrome, STIs: Sexually transmitted infections | | | | | | |
